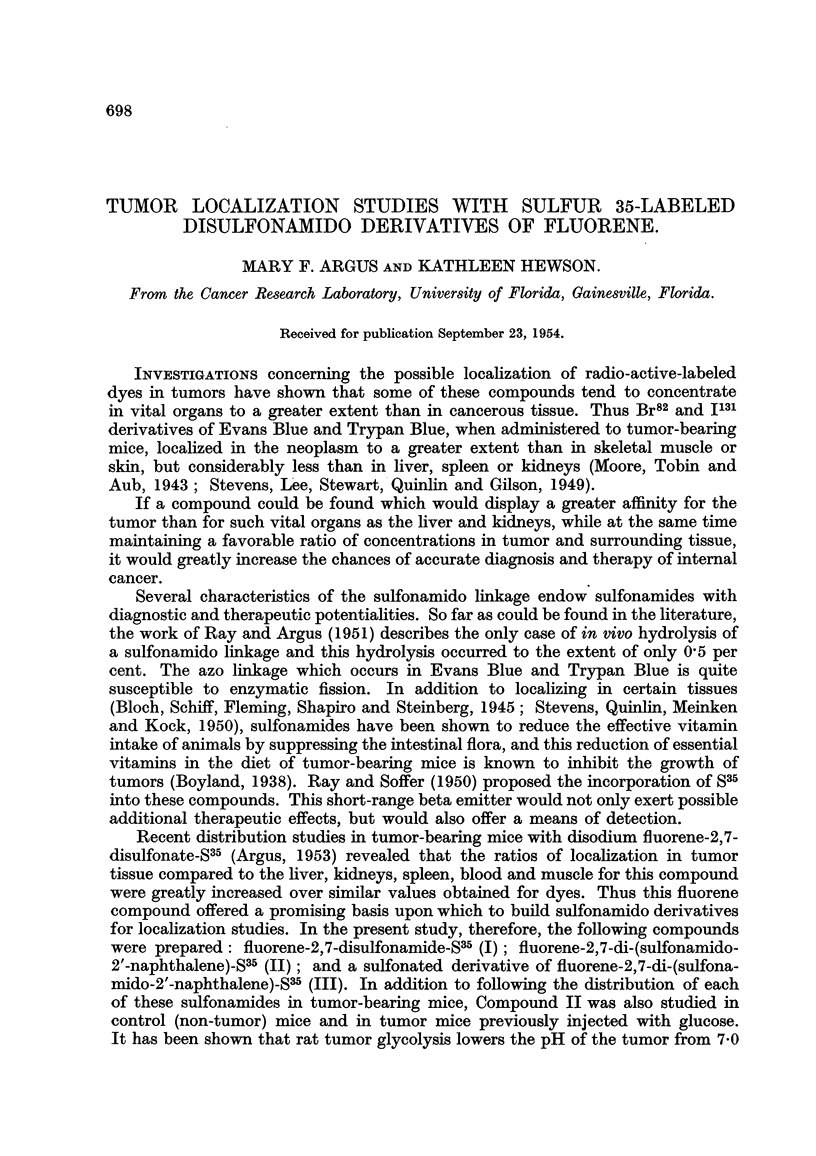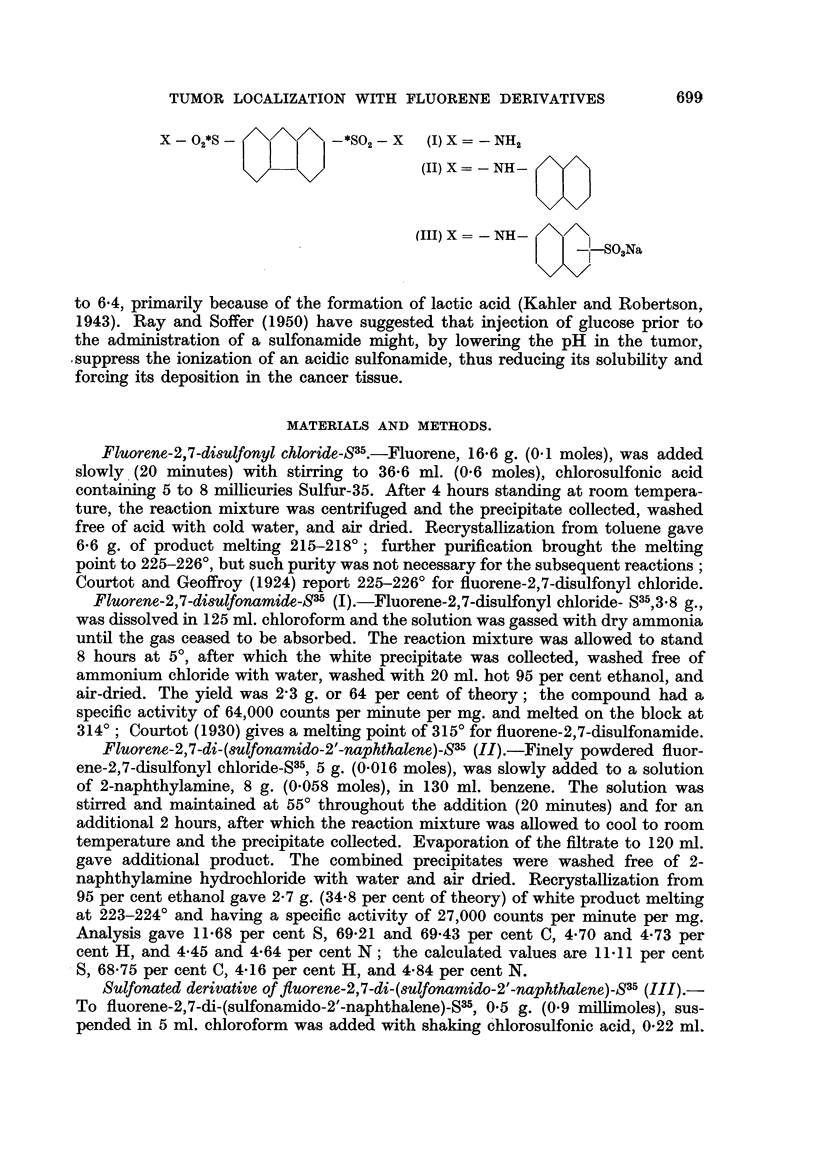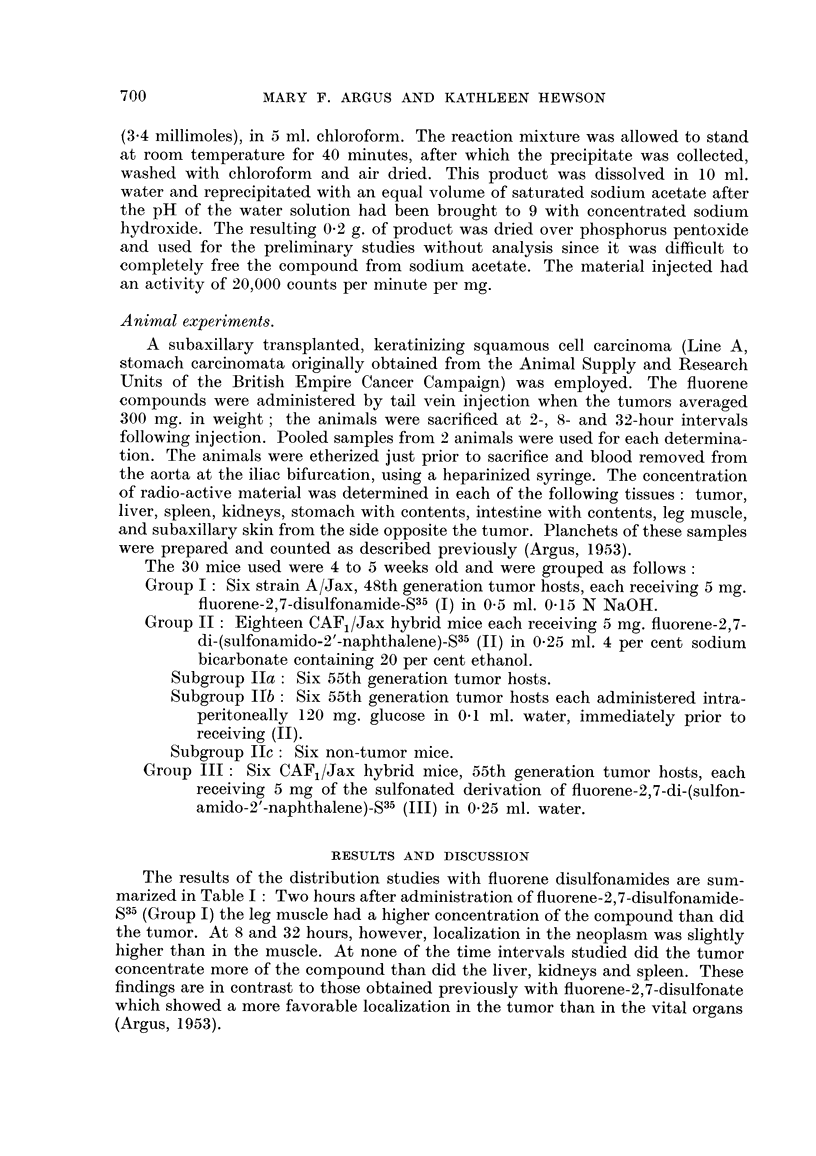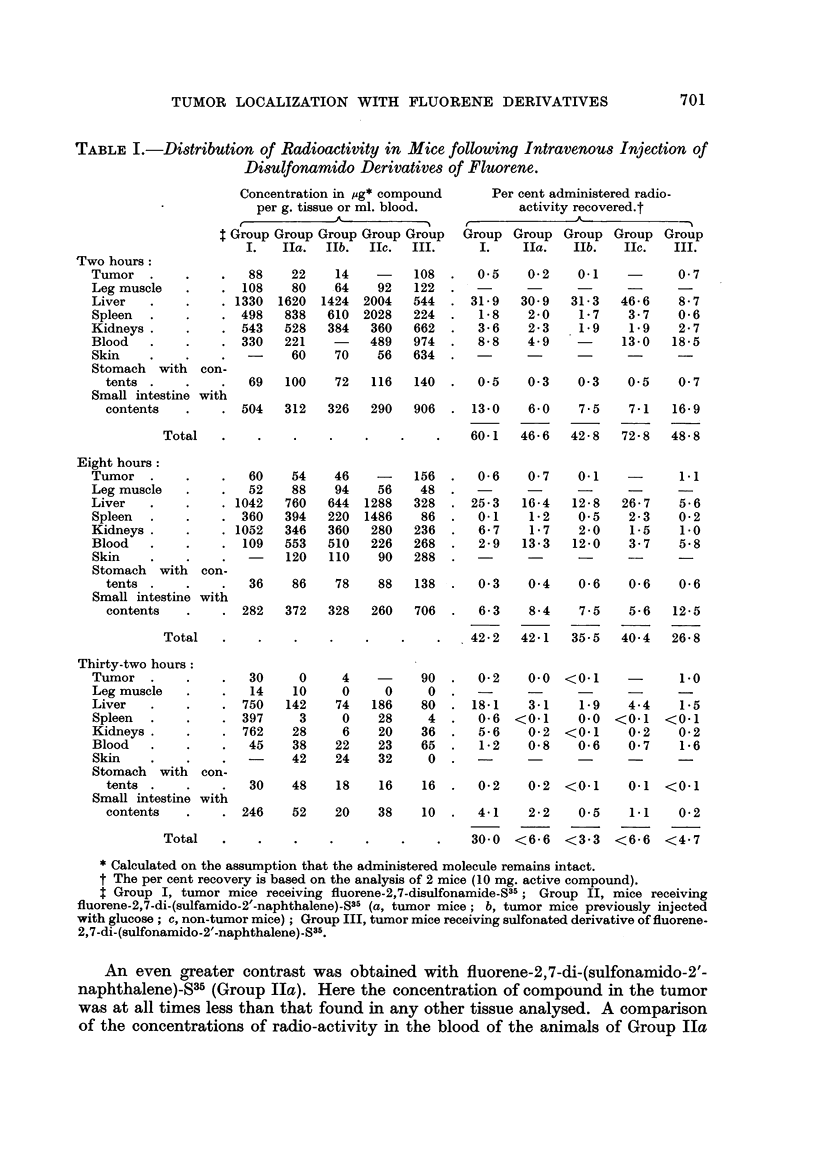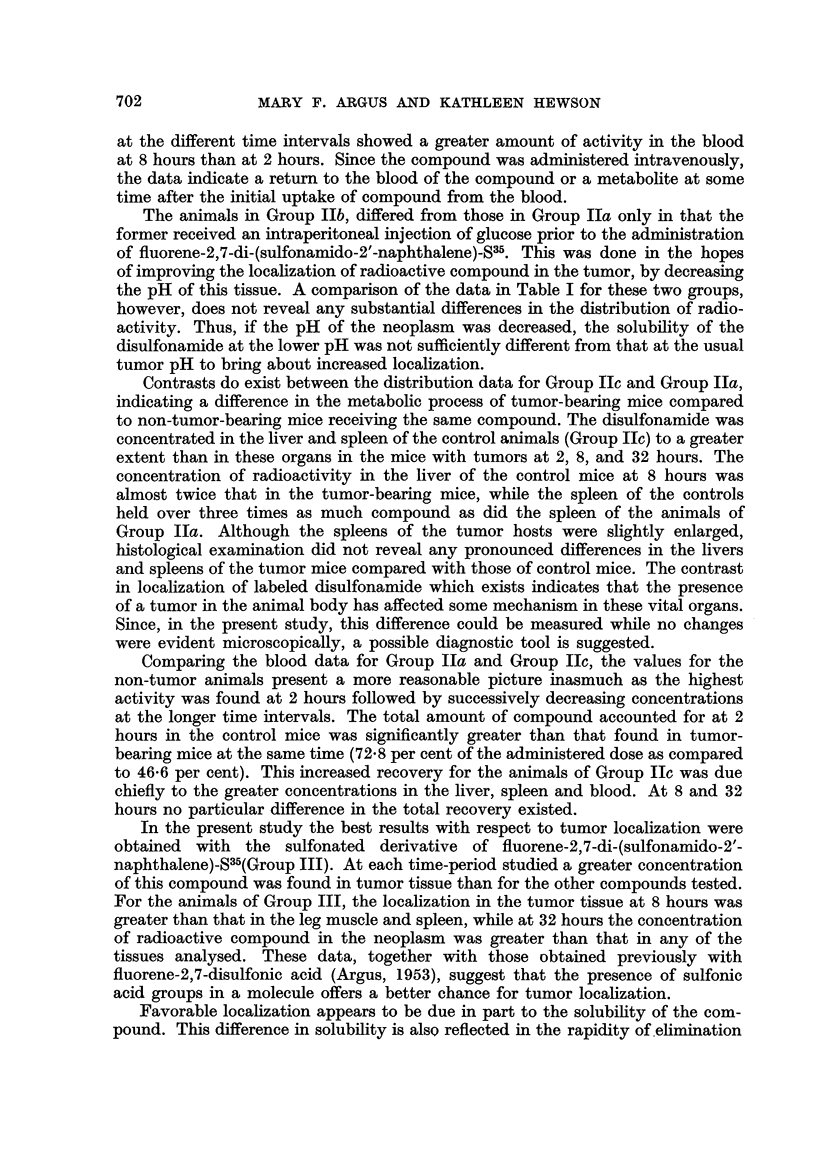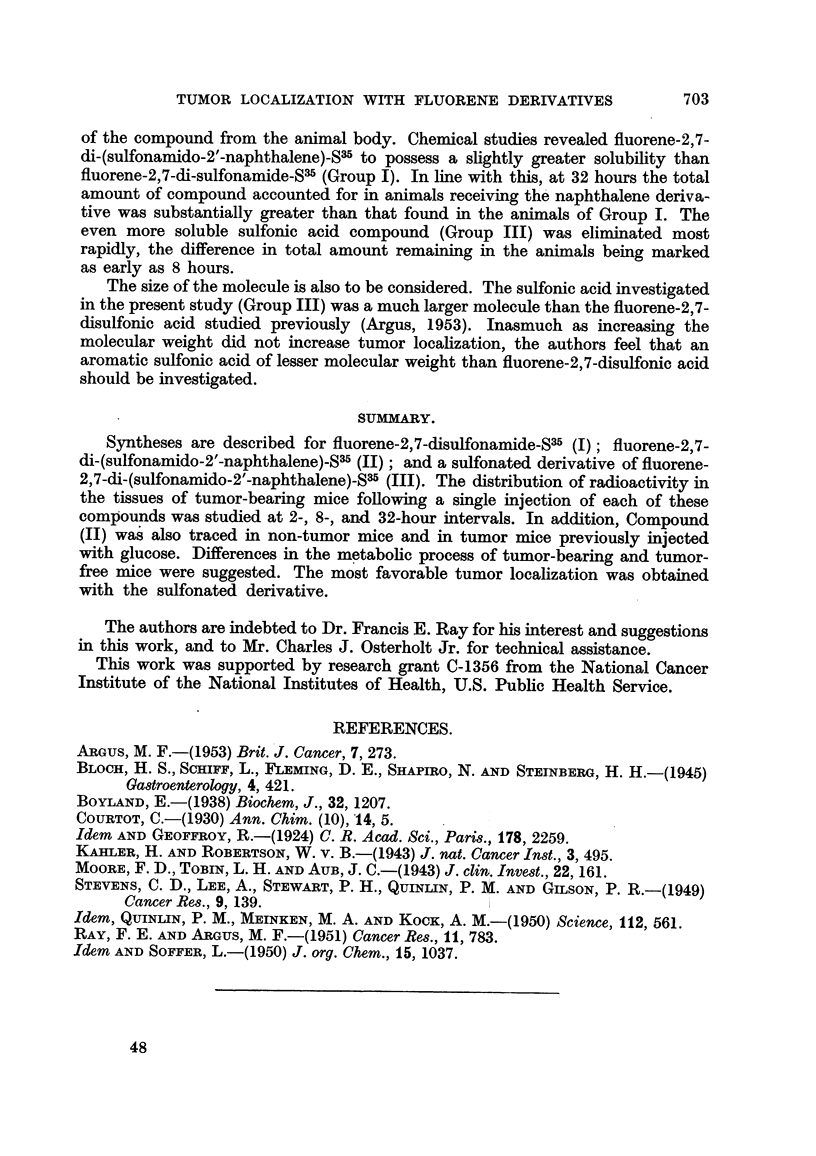# Tumour Localization Studies with Sulfur 35-Labeled Disulfonamido Derivatives of Fluorene

**DOI:** 10.1038/bjc.1954.76

**Published:** 1954-12

**Authors:** Mary F. Argus, Kathleen Hewson


### TUMOR LOCALIZATION STUDIES WITH SULFUR 35-LABELED DISULFONAMIDO DERIVATIVES OF FLUORENE.

MARY F. ARGUS AND KATHLEEN HEWSON.

*From the Cancer Research Laboratory, University of Florida, Gainesville, Florida.*

Received for publication September 23, 1954.

INVESTIGATIONS concerning the possible localization of radio-active-labeled dyes in tumors have shown that some of these compounds tend to concentrate in vital organs to a greater extent than in cancerous tissue. Thus $Br^{82}$ and $I^{131}$ derivatives of Evans Blue and Trypan Blue, when administered to tumor-bearing mice, localized in the neoplasm to a greater extent than in skeletal muscle or skin, but considerably less than in liver, spleen or kidneys (Moore, Tobin and Aub, 1943 ; Stevens, Lee, Stewart, Quinlin and Gilson, 1949).

If a compound could be found which would display a greater affinity for the tumor than for such vital organs as the liver and kidneys, while at the same time maintaining a favorable ratio of concentrations in tumor and surrounding tissue, it would greatly increase the chances of accurate diagnosis and therapy of internal cancer.

Several characteristics of the sulfonamido linkage endow sulfonamides with diagnostic and therapeutic potentialities. So far as could be found in the literature, the work of Ray and Argus (1951) describes the only case of *in vivo* hydrolysis of a sulfonamido linkage and this hydrolysis occurred to the extent of only 0·5 per cent. The azo linkage which occurs in Evans Blue and Trypan Blue is quite susceptible to enzymatic fission. In addition to localizing in certain tissues (Bloch, Schiff, Fleming, Shapiro and Steinberg, 1945 ; Stevens, Quinlin, Meinken and Kock, 1950), sulfonamides have been shown to reduce the effective vitamin intake of animals by suppressing the intestinal flora, and this reduction of essential vitamins in the diet of tumor-bearing mice is known to inhibit the growth of tumors (Boyland, 1938). Ray and Soffer (1950) proposed the incorporation of $S^{35}$ into these compounds. This short-range beta emitter would not only exert possible additional therapeutic effects, but would also offer a means of detection.

Recent distribution studies in tumor-bearing mice with disodium fluorene-2,7-disulfonate-$S^{35}$ (Argus, 1953) revealed that the ratios of localization in tumor tissue compared to the liver, kidneys, spleen, blood and muscle for this compound were greatly increased over similar values obtained for dyes. Thus this fluorene compound offered a promising basis upon which to build sulfonamido derivatives for localization studies. In the present study, therefore, the following compounds were prepared : fluorene-2,7-disulfonamide-$S^{35}$ (I) ; fluorene-2,7-di-(sulfonamido-2′-naphthalene)-$S^{35}$ (II) ; and a sulfonated derivative of fluorene-2,7-di-(sulfonamido-2′-naphthalene)-$S^{35}$ (III). In addition to following the distribution of each of these sulfonamides in tumor-bearing mice, Compound II was also studied in control (non-tumor) mice and in tumor mice previously injected with glucose. It has been shown that rat tumor glycolysis lowers the pH of the tumor from 7·0

to 6·4, primarily because of the formation of lactic acid (Kahler and Robertson, 1943). Ray and Soffer (1950) have suggested that injection of glucose prior to the administration of a sulfonamide might, by lowering the pH in the tumor, suppress the ionization of an acidic sulfonamide, thus reducing its solubility and forcing its deposition in the cancer tissue.

### MATERIALS AND METHODS.

*Fluorene-2,7-disulfonyl chloride-$S^{35}$*.—Fluorene, 16·6 g. (0·1 moles), was added slowly (20 minutes) with stirring to 36·6 ml. (0·6 moles), chlorosulfonic acid containing 5 to 8 millicuries Sulfur-35. After 4 hours standing at room temperature, the reaction mixture was centrifuged and the precipitate collected, washed free of acid with cold water, and air dried. Recrystallization from toluene gave 6·6 g. of product melting 215–218° ; further purification brought the melting point to 225–226°, but such purity was not necessary for the subsequent reactions ; Courtot and Geoffroy (1924) report 225–226° for fluorene-2,7-disulfonyl chloride.

*Fluorene-2,7-disulfonamide-$S^{35}$* (I).—Fluorene-2,7-disulfonyl chloride- $S^{35}$,3·8 g., was dissolved in 125 ml. chloroform and the solution was gassed with dry ammonia until the gas ceased to be absorbed. The reaction mixture was allowed to stand 8 hours at 5°, after which the white precipitate was collected, washed free of ammonium chloride with water, washed with 20 ml. hot 95 per cent ethanol, and air-dried. The yield was 2·3 g. or 64 per cent of theory ; the compound had a specific activity of 64,000 counts per minute per mg. and melted on the block at 314° ; Courtot (1930) gives a melting point of 315° for fluorene-2,7-disulfonamide.

*Fluorene-2,7-di-(sulfonamido-2′-naphthalene)-$S^{35}$ (II)*.—Finely powdered fluorene-2,7-disulfonyl chloride-$S^{35}$, 5 g. (0·016 moles), was slowly added to a solution of 2-naphthylamine, 8 g. (0·058 moles), in 130 ml. benzene. The solution was stirred and maintained at 55° throughout the addition (20 minutes) and for an additional 2 hours, after which the reaction mixture was allowed to cool to room temperature and the precipitate collected. Evaporation of the filtrate to 120 ml. gave additional product. The combined precipitates were washed free of 2-naphthylamine hydrochloride with water and air dried. Recrystallization from 95 per cent ethanol gave 2·7 g. (34·8 per cent of theory) of white product melting at 223–224° and having a specific activity of 27,000 counts per minute per mg. Analysis gave 11·68 per cent S, 69·21 and 69·43 per cent C, 4·70 and 4·73 per cent H, and 4·45 and 4·64 per cent N ; the calculated values are 11·11 per cent S, 68·75 per cent C, 4·16 per cent H, and 4·84 per cent N.

*Sulfonated derivative of fluorene-2,7-di-(sulfonamido-2′-naphthalene)-$S^{35}$ (III)*.—To fluorene-2,7-di-(sulfonamido-2′-naphthalene)-$S^{35}$, 0·5 g. (0·9 millimoles), suspended in 5 ml. chloroform was added with shaking chlorosulfonic acid, 0·22 ml.

(3·4 millimoles), in 5 ml. chloroform. The reaction mixture was allowed to stand at room temperature for 40 minutes, after which the precipitate was collected, washed with chloroform and air dried. This product was dissolved in 10 ml. water and reprecipitated with an equal volume of saturated sodium acetate after the pH of the water solution had been brought to 9 with concentrated sodium hydroxide. The resulting 0·2 g. of product was dried over phosphorus pentoxide and used for the preliminary studies without analysis since it was difficult to completely free the compound from sodium acetate. The material injected had an activity of 20,000 counts per minute per mg.

*Animal experiments.*

A subaxillary transplanted, keratinizing squamous cell carcinoma (Line A, stomach carcinomata originally obtained from the Animal Supply and Research Units of the British Empire Cancer Campaign) was employed. The fluorene compounds were administered by tail vein injection when the tumors averaged 300 mg. in weight ; the animals were sacrificed at 2-, 8- and 32-hour intervals following injection. Pooled samples from 2 animals were used for each determination. The animals were etherized just prior to sacrifice and blood removed from the aorta at the iliac bifurcation, using a heparinized syringe. The concentration of radio-active material was determined in each of the following tissues : tumor, liver, spleen, kidneys, stomach with contents, intestine with contents, leg muscle, and subaxillary skin from the side opposite the tumor. Planchets of these samples were prepared and counted as described previously (Argus, 1953).

The 30 mice used were 4 to 5 weeks old and were grouped as follows :

Group I : Six strain A/Jax, 48th generation tumor hosts, each receiving 5 mg. fluorene-2,7-disulfonamide-$S^{35}$ (I) in 0·5 ml. 0·15 N NaOH.

Group II : Eighteen $CAF_1$/Jax hybrid mice each receiving 5 mg. fluorene-2,7-di-(sulfonamido-2′-naphthalene)-$S^{35}$ (II) in 0·25 ml. 4 per cent sodium bicarbonate containing 20 per cent ethanol.

Subgroup II*a* : Six 55th generation tumor hosts.

Subgroup II*b* : Six 55th generation tumor hosts each administered intraperitoneally 120 mg. glucose in 0·1 ml. water, immediately prior to receiving (II).

Subgroup II*c* : Six non-tumor mice.

Group III : Six $CAF_1$/Jax hybrid mice, 55th generation tumor hosts, each receiving 5 mg of the sulfonated derivation of fluorene-2,7-di-(sulfonamido-2′-naphthalene)-$S^{35}$ (III) in 0·25 ml. water.

### RESULTS AND DISCUSSION

The results of the distribution studies with fluorene disulfonamides are summarized in Table I : Two hours after administration of fluorene-2,7-disulfonamide-$S^{35}$ (Group I) the leg muscle had a higher concentration of the compound than did the tumor. At 8 and 32 hours, however, localization in the neoplasm was slightly higher than in the muscle. At none of the time intervals studied did the tumor concentrate more of the compound than did the liver, kidneys and spleen. These findings are in contrast to those obtained previously with fluorene-2,7-disulfonate which showed a more favorable localization in the tumor than in the vital organs (Argus, 1953).

TABLE I.—*Distribution of Radioactivity in Mice following Intravenous Injection of Disulfonamido Derivatives of Fluorene.*

| | Concentration in $\mu$g* compound per g. tissue or ml. blood. | | | | | Per cent administered radio-activity recovered.† | | | | |
|---|---|---|---|---|---|---|---|---|---|---|
| ‡ | Group I. | Group IIa. | Group IIb. | Group IIc. | Group III. | Group I. | Group IIa. | Group IIb. | Group IIc. | Group III. |
| Two hours : | | | | | | | | | | |
| Tumor | 88 | 22 | 14 | — | 108 | 0·5 | 0·2 | 0·1 | — | 0·7 |
| Leg muscle | 108 | 80 | 64 | 92 | 122 | — | — | — | — | — |
| Liver | 1330 | 1620 | 1424 | 2004 | 544 | 31·9 | 30·9 | 31·3 | 46·6 | 8·7 |
| Spleen | 498 | 838 | 610 | 2028 | 224 | 1·8 | 2·0 | 1·7 | 3·7 | 0·6 |
| Kidneys | 543 | 528 | 384 | 360 | 662 | 3·6 | 2·3 | 1·9 | 1·9 | 2·7 |
| Blood | 330 | 221 | — | 489 | 974 | 8·8 | 4·9 | — | 13·0 | 18·5 |
| Skin | — | 60 | 70 | 56 | 634 | — | — | — | — | — |
| Stomach with contents | 69 | 100 | 72 | 116 | 140 | 0·5 | 0·3 | 0·3 | 0·5 | 0·7 |
| Small intestine with contents | 504 | 312 | 326 | 290 | 906 | 13·0 | 6·0 | 7·5 | 7·1 | 16·9 |
| Total | | | | | | 60·1 | 46·6 | 42·8 | 72·8 | 48·8 |
| Eight hours : | | | | | | | | | | |
| Tumor | 60 | 54 | 46 | — | 156 | 0·6 | 0·7 | 0·1 | — | 1·1 |
| Leg muscle | 52 | 88 | 94 | 56 | 48 | — | — | — | — | — |
| Liver | 1042 | 760 | 644 | 1288 | 328 | 25·3 | 16·4 | 12·8 | 26·7 | 5·6 |
| Spleen | 360 | 394 | 220 | 1486 | 86 | 0·1 | 1·2 | 0·5 | 2·3 | 0·2 |
| Kidneys | 1052 | 346 | 360 | 280 | 236 | 6·7 | 1·7 | 2·0 | 1·5 | 1·0 |
| Blood | 109 | 553 | 510 | 226 | 268 | 2·9 | 13·3 | 12·0 | 3·7 | 5·8 |
| Skin | — | 120 | 110 | 90 | 288 | — | — | — | — | — |
| Stomach with contents | 36 | 86 | 78 | 88 | 138 | 0·3 | 0·4 | 0·6 | 0·6 | 0·6 |
| Small intestine with contents | 282 | 372 | 328 | 260 | 706 | 6·3 | 8·4 | 7·5 | 5·6 | 12·5 |
| Total | | | | | | 42·2 | 42·1 | 35·5 | 40·4 | 26·8 |
| Thirty-two hours : | | | | | | | | | | |
| Tumor | 30 | 0 | 4 | — | 90 | 0·2 | 0·0 | <0·1 | — | 1·0 |
| Leg muscle | 14 | 10 | 0 | 0 | 0 | — | — | — | — | — |
| Liver | 750 | 142 | 74 | 186 | 80 | 18·1 | 3·1 | 1·9 | 4·4 | 1·5 |
| Spleen | 397 | 3 | 0 | 28 | 4 | 0·6 | <0·1 | 0·0 | <0·1 | <0·1 |
| Kidneys | 762 | 28 | 6 | 20 | 36 | 5·6 | 0·2 | <0·1 | 0·2 | 0·2 |
| Blood | 45 | 38 | 22 | 23 | 65 | 1·2 | 0·8 | 0·6 | 0·7 | 1·6 |
| Skin | — | 42 | 24 | 32 | 0 | — | — | — | — | — |
| Stomach with contents | 30 | 48 | 18 | 16 | 16 | 0·2 | 0·2 | <0·1 | 0·1 | <0·1 |
| Small intestine with contents | 246 | 52 | 20 | 38 | 10 | 4·1 | 2·2 | 0·5 | 1·1 | 0·2 |
| Total | | | | | | 30·0 | <6·6 | <3·3 | <6·6 | <4·7 |

* Calculated on the assumption that the administered molecule remains intact.

† The per cent recovery is based on the analysis of 2 mice (10 mg. active compound).

‡ Group I, tumor mice receiving fluorene-2,7-disulfonamide-$S^{35}$ ; Group II, mice receiving fluorene-2,7-di-(sulfamido-2′-naphthalene)-$S^{35}$ (*a*, tumor mice ; *b*, tumor mice previously injected with glucose ; *c*, non-tumor mice) ; Group III, tumor mice receiving sulfonated derivative of fluorene-2,7-di-(sulfonamido-2′-naphthalene)-$S^{35}$.

An even greater contrast was obtained with fluorene-2,7-di-(sulfonamido-2′-naphthalene)-$S^{35}$ (Group II*a*). Here the concentration of compound in the tumor was at all times less than that found in any other tissue analysed. A comparison of the concentrations of radio-activity in the blood of the animals of Group II*a*

at the different time intervals showed a greater amount of activity in the blood at 8 hours than at 2 hours. Since the compound was administered intravenously, the data indicate a return to the blood of the compound or a metabolite at some time after the initial uptake of compound from the blood.

The animals in Group II*b*, differed from those in Group II*a* only in that the former received an intraperitoneal injection of glucose prior to the administration of fluorene-2,7-di-(sulfonamido-2′-naphthalene)-$S^{35}$. This was done in the hopes of improving the localization of radioactive compound in the tumor, by decreasing the pH of this tissue. A comparison of the data in Table I for these two groups, however, does not reveal any substantial differences in the distribution of radioactivity. Thus, if the pH of the neoplasm was decreased, the solubility of the disulfonamide at the lower pH was not sufficiently different from that at the usual tumor pH to bring about increased localization.

Contrasts do exist between the distribution data for Group II*c* and Group II*a*, indicating a difference in the metabolic process of tumor-bearing mice compared to non-tumor-bearing mice receiving the same compound. The disulfonamide was concentrated in the liver and spleen of the control animals (Group II*c*) to a greater extent than in these organs in the mice with tumors at 2, 8, and 32 hours. The concentration of radioactivity in the liver of the control mice at 8 hours was almost twice that in the tumor-bearing mice, while the spleen of the controls held over three times as much compound as did the spleen of the animals of Group II*a*. Although the spleens of the tumor hosts were slightly enlarged, histological examination did not reveal any pronounced differences in the livers and spleens of the tumor mice compared with those of control mice. The contrast in localization of labeled disulfonamide which exists indicates that the presence of a tumor in the animal body has affected some mechanism in these vital organs. Since, in the present study, this difference could be measured while no changes were evident microscopically, a possible diagnostic tool is suggested.

Comparing the blood data for Group II*a* and Group II*c*, the values for the non-tumor animals present a more reasonable picture inasmuch as the highest activity was found at 2 hours followed by successively decreasing concentrations at the longer time intervals. The total amount of compound accounted for at 2 hours in the control mice was significantly greater than that found in tumor-bearing mice at the same time (72·8 per cent of the administered dose as compared to 46·6 per cent). This increased recovery for the animals of Group II*c* was due chiefly to the greater concentrations in the liver, spleen and blood. At 8 and 32 hours no particular difference in the total recovery existed.

In the present study the best results with respect to tumor localization were obtained with the sulfonated derivative of fluorene-2,7-di-(sulfonamido-2′-naphthalene)-$S^{35}$(Group III). At each time-period studied a greater concentration of this compound was found in tumor tissue than for the other compounds tested. For the animals of Group III, the localization in the tumor tissue at 8 hours was greater than that in the leg muscle and spleen, while at 32 hours the concentration of radioactive compound in the neoplasm was greater than that in any of the tissues analysed. These data, together with those obtained previously with fluorene-2,7-disulfonic acid (Argus, 1953), suggest that the presence of sulfonic acid groups in a molecule offers a better chance for tumor localization.

Favorable localization appears to be due in part to the solubility of the compound. This difference in solubility is also reflected in the rapidity of elimination of the compound from the animal body. Chemical studies revealed fluorene-2,7-di-(sulfonamido-2′-naphthalene)-$S^{35}$ to possess a slightly greater solubility than fluorene-2,7-di-sulfonamide-$S^{35}$ (Group I). In line with this, at 32 hours the total amount of compound accounted for in animals receiving the naphthalene derivative was substantially greater than that found in the animals of Group I. The even more soluble sulfonic acid compound (Group III) was eliminated most rapidly, the difference in total amount remaining in the animals being marked as early as 8 hours.

The size of the molecule is also to be considered. The sulfonic acid investigated in the present study (Group III) was a much larger molecule than the fluorene-2,7-disulfonic acid studied previously (Argus, 1953). Inasmuch as increasing the molecular weight did not increase tumor localization, the authors feel that an aromatic sulfonic acid of lesser molecular weight than fluorene-2,7-disulfonic acid should be investigated.

### SUMMARY.

Syntheses are described for fluorene-2,7-disulfonamide-$S^{35}$ (I); fluorene-2,7-di-(sulfonamido-2′-naphthalene)-$S^{35}$ (II); and a sulfonated derivative of fluorene-2,7-di-(sulfonamido-2′-naphthalene)-$S^{35}$ (III). The distribution of radioactivity in the tissues of tumor-bearing mice following a single injection of each of these compounds was studied at 2-, 8-, and 32-hour intervals. In addition, Compound (II) was also traced in non-tumor mice and in tumor mice previously injected with glucose. Differences in the metabolic process of tumor-bearing and tumor-free mice were suggested. The most favorable tumor localization was obtained with the sulfonated derivative.

The authors are indebted to Dr. Francis E. Ray for his interest and suggestions in this work, and to Mr. Charles J. Osterholt Jr. for technical assistance.

This work was supported by research grant C-1356 from the National Cancer Institute of the National Institutes of Health, U.S. Public Health Service.

### REFERENCES.

Argus, M. F.—(1953) *Brit. J. Cancer*, **7**, 273.

Bloch, H. S., Schiff, L., Fleming, D. E., Shapiro, N. and Steinberg, H. H.—(1945) *Gastroenterology*, **4**, 421.

Boyland, E.—(1938) *Biochem, J.*, **32**, 1207.

Courtot, C.—(1930) *Ann. Chim.* (10), **14**, 5.

*Idem* and Geoffroy, R.—(1924) *C. R. Acad. Sci., Paris.*, **178**, 2259.

Kahler, H. and Robertson, W. v. B.—(1943) *J. nat. Cancer Inst.*, **3**, 495.

Moore, F. D., Tobin, L. H. and Aub, J. C.—(1943) *J. clin. Invest.*, **22**, 161.

Stevens, C. D., Lee, A., Stewart, P. H., Quinlin, P. M. and Gilson, P. R.—(1949) *Cancer Res.*, **9**, 139.

*Idem*, Quinlin, P. M., Meinken, M. A. and Kock, A. M.—(1950) *Science*, **112**, 561.

Ray, F. E. and Argus, M. F.—(1951) *Cancer Res.*, **11**, 783.

*Idem* and Soffer, L.—(1950) *J. org. Chem.*, **15**, 1037.